# Population genomics and conservation management of the threatened black-footed tree-rat (*Mesembriomys gouldii*) in northern Australia

**DOI:** 10.1038/s41437-023-00601-0

**Published:** 2023-03-10

**Authors:** Brenton von Takach, Holly Sargent, Cara E. Penton, Kate Rick, Brett P. Murphy, Georgina Neave, Hugh F. Davies, Brydie M. Hill, Sam C. Banks

**Affiliations:** 1 School of Molecular and Life Sciences, Curtin University, Perth, WA Australia; 2 Research Institute for the Environment and Livelihoods, Charles Darwin University, Darwin, NT 0909 Australia; 3 Warddeken Land Management Ltd, Darwin, NT Australia; 4 School of Biological Sciences, The University of Western Australia, Crawley, WA 6009 Australia; 5 Flora and Fauna Division, Department of Environment, Parks and Water Security, Northern Territory Government, Berrimah, NT 0831 Australia

**Keywords:** Ecological genetics, Molecular ecology, Population genetics, Conservation biology

## Abstract

Genomic diversity is a fundamental component of Earth’s total biodiversity, and requires explicit consideration in efforts to conserve biodiversity. To conserve genomic diversity, it is necessary to measure its spatial distribution, and quantify the contribution that any intraspecific evolutionary lineages make to overall genomic diversity. Here, we describe the range-wide population genomic structure of a threatened Australian rodent, the black-footed tree-rat (*Mesembriomys gouldii*), aiming to provide insight into the timing and extent of population declines across a large region with a dearth of long-term monitoring data. By estimating recent trajectories in effective population sizes at four localities, we confirm widespread population decline across the species’ range, but find that the population in the peri-urban area of the Darwin region has been more stable. Based on current sampling, the Melville Island population made the greatest contribution to overall allelic richness of the species, and the prioritisation analysis suggested that conservation of the Darwin and Cobourg Peninsula populations would be the most cost-effective scenario to retain more than 90% of all alleles. Our results broadly confirm current sub-specific taxonomy, and provide crucial data on the spatial distribution of genomic diversity to help prioritise limited conservation resources. Along with additional sampling and genomic analysis from the far eastern and western edges of the black-footed tree-rat distribution, we suggest a range of conservation and research priorities that could help improve black-footed tree-rat population trajectories at large and fine spatial scales, including the retention and expansion of structurally complex habitat patches.

## Introduction

The Earth is experiencing its sixth mass extinction event (Leakey and Lewin 1995; Ceballos et al. 2015; Díaz et al. 2019), with severe declines of many species driven by pervasive anthropogenic disturbance including habitat degradation, invasive species, land clearing and overharvesting (Cardillo et al. 2008; Schipper et al. 2008). While the loss of native species is increasingly apparent, another more subtle reduction in biodiversity is occurring through the erosion of genomic diversity within species (Roycroft et al. 2021). Genomic diversity is increasingly being recognised as a fundamental component of the Earth’s total biodiversity (Hoban et al. 2020). Hence, the post-2020 Global Biodiversity Framework of the UN Convention on Biological Diversity aims to safeguard genomic diversity of wild and domesticated species, “with at least 90 percent of genetic diversity within all species maintained” (CBD 2021).

To achieve this, the spatial distribution of such diversity needs to be measured, with the contribution that various evolutionary lineages or populations make to overall diversity within species quantified. Analogous to how a sound understanding of species boundaries is critical for the conservation of species diversity, a sound understanding of inter- and intraspecific population genomic structure is necessary to conserve genomic diversity. Genomic analyses can provide improved understanding of the taxonomic identity of populations and species, quantify the loss of genomic diversity from populations, and deliver information critical to the success of genetic rescue, reintroduction, or translocation strategies (Ottewell et al. 2016).

With at least 33 species of native mammals driven to extinction since European colonisation of the country in 1788, Australia has the highest mammal extinction rate globally (Woinarski et al. 2019; Roycroft et al. 2021). Declines in mammal richness and abundance are also continuing, suggesting further extinctions are likely (Woinarski et al. 2010; Davies et al. 2018). Rodents have been particularly susceptible to this process; while they make up about 19% of the Australian mammal fauna (Van Dyck et al. 2013), they represent 39% of mammal extinctions since European colonisation (Smith and Quin 1996; Woinarski et al. 2019). Understanding the broad patterns, causes and consequences of these declines and extinctions are vital for conservation policy and practice (Amori and Gippoliti 2003). One rodent species currently declining across northern Australia is the black-footed tree-rat (*Mesembriomys gouldii*), a semi-arboreal rodent that has experienced an estimated 30–50% decline in population size throughout the 2000s (Woinarski et al. 2014). A recent study in the Northern Territory found a 33% reduction in geographic range and a 46% reduction in niche breadth (von Takach et al. 2020a, 2020b). The species is now listed as Vulnerable on the IUCN Red List (Woinarski and Burbidge 2016), and is variously listed as threatened under state, territory, and federal Australian legislation (Supplementary Material Table S1).

**Fig. 1:**
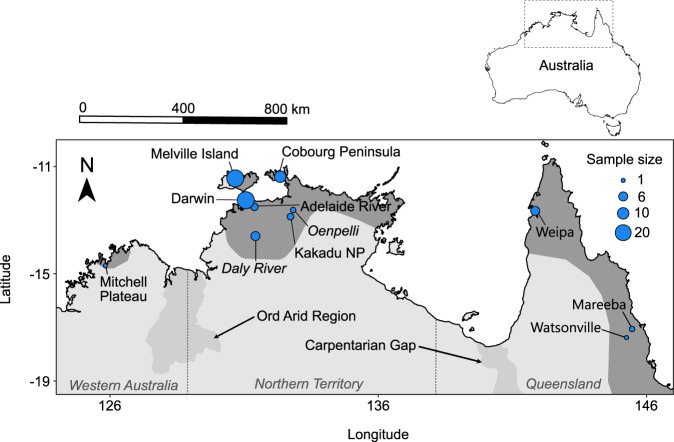
The locations (blue points) of all black-footed tree-rat (*Mesembriomys gouldii*) tissue samples collected for population genomic analysis. Points are sized are relative to the number of samples collected from each locality. The dark grey shading indicates the current range of the black-footed tree-rat (Woinarski and Burbidge 2016). Light grey shading represents biogeographic barriers that are thought to drive population structure in other taxa. Note that the samples from the Oenpelli and Daly River localities (italicised) were removed during filtering due to low data quality.

Three subspecies of the black-footed tree-rat have been recognised, based on minor morphological differences in skull and foot shape, as well as variation in colouration. These include *M. g. gouldii* from the central and western mainland components of the species range; *M. g. melvillensis* from an offshore island; and *M. g. rattoides* from the eastern portion of the species range (Fig. 1) (Troughton 1967; Van Dyck and Strahan 2008). These subspecies broadly conform to major biogeographic barriers that separate the Queensland and north-western Australian populations, and Melville Island and mainland populations. The effects of such barriers on gene flow varies considerably amongst taxa, and genomic analysis helps to clarify the extent to which they impact population genetic differentiation (Melville et al. 2011; Eldridge et al. 2012; Catullo et al. 2014; Edwards et al. 2017; von Takach et al. 2021; von Takach et al. 2022). Such analyses refine our understanding of the geographic locations of evolutionary lineages and clarifies whether such lineages conform to current taxonomic nomenclature based on morphological study.

Here, we generate the first population genomic dataset across the range of the black-footed tree-rat, aiming to improve our understanding of genomic variation and recent population trajectories across its known distribution. We provide information on genomic variation within and among populations and putative lineages to inform conservation actions. We use DNA sequencing data associated with double-digest restriction sites to obtain thousands of genome-wide single-nucleotide polymorphisms (SNPs), and apply a range of analyses to identify patterns of genomic differentiation, population genomic structure, and historical demography. We also use a set of analyses to inform strategies for conserving the genomic diversity of the species. This includes comparison of levels of genetic diversity (heterozygosity and allelic richness) and recent trends in effective population size which may indicate which populations are most at-risk from loss of genomic variation and are potentially candidates for management. It also includes a quantitative prioritisation analysis to identify the most important populations for conservation of the species’ genomic diversity and to quantify the contribution of each sampled population to the species’ genomic diversity. Conservation planning considers ecological, social, cultural and economic factors in addition to genomic data, but our strategy here is to present the genomic analyses independent of any other considerations or constraints.

We hypothesise that (1) multiple evolutionary lineages will be present in this species, likely representing differentiation between mainland populations and offshore islands, and (2) conservation management will need to consider multiple populations and lineages in order to conserve >90% of the genomic diversity of the black-footed tree rat as a species. We also suggest that demographic trajectories inferred from genome-wide SNP data will broadly reflect observations of decline severity from ecological research, while also providing further insight into the timing and extent of population declines.

## Materials and methods

### Study species

The black-footed tree-rat is the second largest Australian rodent (adult body mass ~880 g), inhabiting tropical open forests and woodlands across the northern end of the continent (Friend 1987). The species is primarily frugivorous but supplements its diet with invertebrates, flowers, and grass seeds (Morton 1992; Rankmore and Friend 2008). Individuals den in large tree hollows (predominantly in *Eucalyptus tetrodonta* and *E. miniata*), crowns of the arborescent monocotyledon *Pandanus spiralis* (Penton et al. 2020; Penton et al. 2021), and sometimes in the roofs of buildings (Wheeler 1982). The black-footed tree-rat has the longest gestation period (43–44 days) (Crichton 1969) and smallest litter size (1–3 young) of any Australian rodent. Potential drivers of black-footed tree-rat population declines have been identified as inappropriate fire regimes, predation by feral cats (*Felis catus*), and habitat degradation by feral herbivores (Davies et al. 2017; von Takach et al. 2020a, 2020b; Stobo-Wilson et al. 2020). While much of our understanding of the species’ ecology is based on observations from the Northern Territory, comparatively little is known of the Queensland and Western Australian populations (Fig. 1).

### Sample collection

We assembled a collection of 83 tissue samples from 11 distinct localities across the distribution of the black-footed tree-rat (Fig. 1). Samples were collated from our own live trapping in the Northern Territory (Darwin and Cobourg Peninsula localities) as well as tissues from field ecologists working across northern Australia and museum collections (Museum and Art Gallery of the Northern Territory, South Australian Museum, and Queensland Museum). Trapping efforts within a locality were variable among regional jurisdictions and field teams, but typically involved sampling a set of several small grids (<1 ha) of cage traps spread over 10 to 20 km. All samples taken from the set of grids within a locality were given an identifying name for population genomic analysis. Of the 83 samples, 75 were ear tissue, six were liver, and two were muscle. The maximum pairwise geographic distance between all sampled individuals was 2122 km.

### DNA extraction, library preparation and sequencing

Tissue samples were prepared and extracted in plate format (Qiagen DNeasy 96 Blood & Tissue Kit) following the manufacturer’s protocol with an extended lysis (incubation at 56 °C for 2 h then reduced to 37 °C overnight). Double-stranded DNA concentrations were quantified using a Qubit 3.0 Fluorometer and normalised to 200 ng DNA in 25 µL, and samples arranged on a 96-well plate that included 12 technical replicates and a negative control. The plate was sent for double‐digest restriction‐associated DNA (ddRAD) sequencing at the Australian Genome Research Facility in Melbourne, Victoria (Peterson et al. 2012). An optimal combination of two restriction enzymes was determined using three establishment samples (broadly representative of the species’ distribution), with *Pst*I and *HpyCH4*IV considered most suitable for achieving the best level of amplification and minimising repetitive sequences. As per von Takach et al. (2021), the library preparation protocol consisted of (1) digestion using *Pst*I and *HpyCH4*IV, (2) ligation with one of 48 unique inline barcoded adapters compatible with the restriction site overhang, (3) manual sample pooling, (4) DNA purification (QIAquick PCR Purification Kit followed by SPRIselect paramagnetic beads), (5) size-selection targeting fragments of 280–375 bp in size (BluePippin, Sage Science), and (6) a PCR amplification step where one of two multiplexing index primers was added. Indexed libraries were pooled together and loaded onto flow cells for 150-bp single-end sequencing on an Illumina NextSeq 500 platform.

### Bioinformatics pipeline and SNP filtering

We obtained 326.5 million raw sequence reads from the sequencing platform, of which 314.7 million (96.4%) were retained after using the *process_radtags* function of the stacks software package (Catchen et al. 2013) to demultiplex samples and trim reads to 125 bp (*Phred* quality score ≥ 30). Reads were aligned to the broad-toothed rat (*Mastacomys fuscus*) chromosome-length genome assembly (https://www.dnazoo.org/assemblies/Mastacomys_fuscus) using the bwa (v0.7.17) *mem* algorithm (Li 2013). *Mastacomys* is a species of ‘old endemic’ conilurine rodent closely related to our study species of *Mesembriomys*, although it is nested within the larger *Pseudomys* genus and the name will likely be updated accordingly (Rowe et al. 2008; Roycroft et al. 2020). The resulting sequence alignment/map file for each individual was converted to a binary alignment/map (BAM) file using samtools v1.7-1 (Li et al. 2009). Also using samtools, unmapped reads were filtered out of each BAM file, reads were sorted by scaffold number and position, and the BAM file was indexed.

We used the angsd v0.93 software package (Korneliussen et al. 2014) to perform initial filtering to identify biallelic single-nucleotide polymorphisms (SNPs) and create a SNP-by-sample matrix. Reads were only used to call SNPs if the map quality was ≥ 20 (thus excluding repeat regions of the genome), and loci only retained if they were (1) polymorphic based on a likelihood ratio test *p* ≤ 1 × 10^−5^ (Kim et al. 2011), (2) were genotyped in at least 25% of individuals, (3) had a ≥ 10 reads per locus per sample, and (4) ≤150 reads per locus per sample. Genotypes were called using posterior probabilities assuming a uniform prior, with a posterior probability threshold of at least 0.98 (using GATK genotype likelihoods). This process identified a total of 188,649 SNPs, with a mean read depth per SNP per sample of 25.2, and a median read depth per SNP per sample of 16.9. The SNP-by-sample matrix was then read into the statistical analysis software R v4.1.0 (R Core Team 2021) for all remaining analyses (von Takach et al. 2020a, 2020b). Filters applied in R included removal of SNPs where (1) the proportion of samples in which loci were genotyped was less than 90%, (2) had a minor allele count of less than three, and (3) had an observed heterozygosity (*H*_O_) > 0.6 (to exclude potentially erroneously merged reads) (Supplementary Material Table S2).

To identify any potential for bias in results due to analyses involving closely related individuals, we assessed pairwise relatedness between individuals at each locality. We estimated kinship coefficients using a method-of-moments technique (Weir and Goudet 2017; Goudet et al. 2018), implemented in the ‘hierfstat’ package (Goudet 2005). Two individuals from the Melville Island population were removed due to a high level of relatedness (kinship coefficients > 0.25).

To remove SNPs in linkage disequilibrium, we used the *snpgdsLDpruning* function of the ‘SNPRelate’ package (Zheng et al. 2012). One of each SNP pair was removed if they had a correlation of >0.5 within a sliding window of 100,000 base-pairs, which is the distance at which the 95th percentile of *r*^2^ in wild populations of the house mouse (*Mus musculus*) falls to less than 0.4 (Laurie et al. 2007). Finally, samples missing more than 25% of genotype calls were removed, retaining a total of 4764 SNPs and 48 unique samples for analysis (i.e., not including technical replicates). The overall level of missing data for the filtered SNP-by-sample matrix was 2.5%. None of the retained SNPs were identified as being sex-linked using the custom function produced by Robledo-Ruiz et al. (2022). Note that this set of SNPs, filtered on linkage disequilibrium, is different to that used by *SNeP* for assessing trends in population sizes (described below), as *SNeP* uses linkage-based methods for estimating effective population size.

To determine whether loci showing a putative signal of selection were likely to be influencing population genomic trends, we checked for the presence of outlier loci using two methods. First, we calculated an *F*_ST_ statistic from an individual ancestry matrix and identified outliers using the approach implemented in the LEA package (Frichot and François 2015; Caye et al. 2016; Martins et al. 2016). Second, we used the OutFLANK method, which compares candidate loci to the null distribution of *F*_ST_ to identify SNPs experiencing spatially heterogeneous selection (Whitlock and Lotterhos 2015). Argument values used in OutFLANK included left and right trim fractions of 0.05, minimum expected heterozygosity of 0.05, and a q threshold (false discovery rate) of 0.05. As no outlier loci were discovered using either of the two approaches, we assumed that SNPs experiencing balancing selection or spatially diversifying selection were unlikely to be having a strong influence on the observed patterns. While additional methods of identifying loci under selection could no doubt be applied, factors such as the geographic arrangement, spatial scale, and number of samples can substantially influence findings (von Takach et al. 2021), and understanding patterns of selection is not a primary aim of this study.

Finally, to ensure that relationships between individuals and localities could be accurately inferred from the dataset, we produced a hierarchical clustering dendrogram based on genetic distance, with visual examination of the dendrogram confirming that technical replicates were closely paired together (Supplementary Material Fig. S1). Technical replicates were then removed from the dataset.

### Population genomic diversity and structure

We calculated mean values of genomic diversity metrics for each locality that had at least five individuals retained after filtering, including the number of alleles (*A*), effective number of alleles (*A*_E_), SNP observed heterozygosity (*H*_O_), SNP expected heterozygosity (*H*_E_), and Wright’s fixation index (*F*_IS_). All calculations were made using the ‘gstudio’ package (Dyer 2016) with the inbuilt small sample size correction for heterozygosity calculation. As heterozygosity estimates based on filtered SNP data can show biases due to sample sizes and filtering parameters, we also calculated observed and expected values of autosomal heterozygosity for each locality using the methods of Schmidt et al. (2021), which considers both monomorphic and polymorphic nucleotides. This included building aligned sequences into a STACKS catalogue via the ‘ref_map’ pipeline, using the filtered and sorted BAM files as inputs (using only individuals retained from the previous filtering steps), and analysing the dataset using the core program ‘Populations’ with all missing sites removed. The heterozygosity estimates and standard errors in the subsection of the summary output titled ‘# All positions (variant and fixed)’ were recorded.

We visualised population genomic structure using an individual-level principal coordinate plot of the first two principal coordinate dimensions of a genetic distance matrix. The proportion of variance explained by each axis was also recorded. Genetic distances were calculated using the *prevosti.dist* function of the ‘poppr’ package (Kamvar et al. 2014). To quantify the extent of isolation among localities, we calculated pairwise genomic differentiation via *F*_ST_ values (Weir and Cockerham 1984) using the ‘StAMPP’ package (Pembleton et al. 2013), with 1000 bootstraps to estimate significance values.

To identify patterns of hierarchical population structuring, we used the cross-entropy methods of the ‘tess3r’ package (Caye et al. 2016, 2018). This package applies a model of genomic structure featuring a discrete number (*k*) of ancestral populations, allowing for independent investigation of values for *k* using cross-entropy metrics. It also incorporates the spatial location of sampling, to remove bias associated with patterns of isolation-by-distance. Cross-entropy criteria were calculated for values of *k* between one and 10, and a cross-entropy scree-plot was output for visual interpretation. The matrices of individual admixture coefficients were then extracted and plotted as stacked bar plots to visualise hierarchical population structure.

### Demographic history and trends in effective population size

We explored the historical demographic relationships among our set of populations using the TreeMix v1.13 software package (Pickrell and Pritchard 2012) and all 4764 SNPs to build a maximum likelihood tree that allows both population splits and migration events. This reveals if population splits and divergence are consistent with our observed population structure, as well as tests for the extent of genetic drift and presence of gene flow. TreeMix uses allele frequencies to infer the structure of an unrooted maximum likelihood tree with a stepwise likelihood procedure to test the effect of migration on the residual covariance (Pickrell and Pritchard 2012). We inferred a topology without admixture, allowing up to 10 migration events, with each event repeated 10 times. We inferred the optimal number of migration events (m) by selecting a model that explains at least 99.8% of variation, as recommended by Pickrell and Pritchard (2012), as well as the second-order rate of change in likelihood (Δm) across incremental values of m using the R package ‘OptM’ (Fitak 2021).

We investigated recent changes in effective population size (*N*_e_) to identify patterns of historical demography in each locality. The *SNeP* v1.1 software tool (Barbato et al. 2015) was used to estimate recent *N*_e_ trends based on the relationship between linkage disequilibrium (LD) and *N*_e_ (Corbin et al. 2012). The variant call format (VCF) file output from angsd was split into individual VCF files for each locality, with plink v1.9 (Purcell et al. 2007) used to generate input files for *SNeP*. The total number of SNPs provided to *SNeP* was thus 188,649, with the software removing any SNPs missing genotypes independently for each locality and a minor allele threshold of 0.05. This retained a variable number of SNPs, depending on the genomic scaffold and the locality being analysed, with typically > 5000 SNPs on the largest scaffolds. We used the Sved and Feldman (1973) mutation rate modifier for correcting the default recombination rate and a sample size correction for unphased genotypes. To investigate uncertainty in demographic trajectories, we bootstrapped the data by removing 10% of individuals from each locality and running *SNeP* 10 times on subsets of samples (five times for Weipa due to the lower sample size at this locality). Effective population sizes for the past 100 years were plotted for each locality using the *geom_smooth* function of the ‘ggplot2’ package (Wickham 2009) and the *x*-axis scaled to time, assuming a mean generation time of 1.95 years (Pacifici et al. 2013).

### Identifying population-level contributions to species genomic diversity

We conducted a set of analyses to identify the contribution of the sampled populations to genomic diversity in the black-footed tree-rat across northern Australia, as has been done for the brush-tailed rabbit-rat (*Conilurus penicillatus*) (von Takach et al. 2021). We focussed on allelic richness (the number of alleles per population, with standardisation for sample size) as this metric is considered a good indicator of evolutionary potential (Caballero and García-Dorado 2013; Greenbaum et al. 2014). An individual population can contribute to genetic diversity of a species through its genetic diversity and through its complementarity, or uniqueness, relative to the other populations in the set (Petit et al. 1998). First, we used the approach of Petit et al. (1998) to quantify the contribution of each locality to the total allelic richness represented across the 4764 SNP panel and the entire set of localities sampled. As we only had a single sample from the Kimberley region, where the black-footed tree-rat is geographically restricted and very rarely recorded (e.g., no records between 1987 and 2020), we excluded this area from the analysis. We used the *allel.rich* function of the ‘PopGenReport’ package (Adamack and Gruber 2014) to estimate mean allelic richness per locus over the entire dataset, standardised to a sample size of 10 alleles to account for differences in sample sizes. We then iteratively removed each locality from the dataset to estimate the proportional loss of allelic richness that would result from extinction of any one of these localities. The contribution of a locality to AR was given by the formula AR(*t*) – AR(-*i*) / (AR(*t*) – 1), where AR(*t*) is total allelic richness and AR(-*i*) is allelic richness over all localities excluding the one in question.

Second, we used marxan (Ball et al. 2009; Watts et al. 2009) to identify networks of extant populations that would best represent/conserve genomic diversity in the species, as estimated by the sampled localities and across all SNP loci. In the absence of specific costed conservation options, we allocated an equal unit cost of 1 to conserve each locality and identified the optimal network of localities to maximise allelic richness in the species, identifying optimal solutions for scenarios of one, two, three, or four ‘protected’ localities using the R package ‘prioritizr’ (Hanson et al. 2020) and the symphony integer linear programming solver (Vladislav 2018). Using this method, each allele is considered a feature to be conserved, and each locality is considered a planning unit. For each of 100 iterations, we randomly sampled four individuals per locality, calculated allelic richness and the total number of alleles across all localities combined and identified a conservation solution for a maximum coverage (of alleles) objective for budgets of 1, 2, 3, and 4. We tallied the number of configurations across the 100 replicates for each budget, as well as the resulting allelic richness and total allele count for each solution.

## Results

### Population genetic diversity and structure

We observed small differences between the patterns of SNP and autosomal heterozygosity estimates (Table 1). Assuming that the autosomal heterozygosity metric is most appropriate for interpretation, we found the highest levels of genomic diversity in the Cobourg Peninsula (*H*_E_ auto = 0.62) and Melville Island (*H*_E_ auto = 0.54) populations, and lowest levels in the Darwin (*H*_E_ = 0.49) and Weipa (*H*_E_ auto = 0.41) populations (Table 1). Importantly, we found high values of both SNP and autosomal heterozygosity in the Cobourg Peninsula population, and the lowest values of both metrics in the Weipa population. Values of *F*_IS_, which measures the excess or deficit of heterozygotes relative to population-level expectations, were positive and close to zero in most cases (*F*_IS_ = 0.01–0.04), demonstrating a slight deficit of heterozygotes at most localities. However, the value for the Weipa population was negative (*F*_IS_ = −0.04), demonstrating a slight excess of heterozygotes.

**Table 1:** Population genomic metrics and standard errors (SE) for black-footed tree-rat (*Mesembriomys gouldii*) localities where *n* ≥ 5.

Pop	n	*A*	*A*_E_	*H*_E_	*H*_O_	*H*_E_ auto	*H*_O_ auto	*F*_IS_
Weipa	5	1.302	1.179	0.118	0.124	0.410	0.460	−0.037
SE		0.007	0.005	0.003	0.003	<0.001	<0.001	0.008
Melville Island	14	1.411	1.211	0.131	0.128	0.540	0.550	0.023
SE		0.007	0.005	0.003	0.003	<0.001	<0.001	0.006
Cobourg Peninsula	8	1.509	1.255	0.166	0.165	0.620	0.650	0.007
SE		0.007	0.005	0.003	0.003	<0.001	<0.001	0.006
Darwin	14	1.620	1.261	0.17	0.161	0.490	0.480	0.044
SE		0.007	0.005	0.003	0.003	<0.001	<0.001	0.005

Metrics include the sample size (*n*), mean number of alleles (*A*), effective number of alleles (*A*_E_), SNP expected heterozygosity (*H*_E_), SNP observed heterozygosity (*H*_O_), autosomal expected heterozygosity (*H*_E_ auto), autosomal observed heterozygosity (*H*_O_ auto), and Wright’s fixation index (*F*_IS_). Note that the autosomal heterozygosity values have been multiplied by 1000 for ease of comparison.

Pairwise genomic differentiation was greatest between Melville Island and Weipa (*F*_ST_ = 0.37). Substantially lower values were found among mainland localities, although the Weipa population was strongly differentiated from populations in the Northern Territory (*F*_ST_ = 0.25–0.28). Localities within the Northern Territory showed considerably less differentiation (*F*_ST_ = 0.13) (Table 2).

**Table 2:** Pairwise genomic differentiation (*F*_ST_) between all populations of black-footed tree-rat (*Mesembriomys gouldii*) with ≥5 samples.

	Weipa	Melville Island	Cobourg Peninsula	Darwin
Weipa	0	<0.01	<0.01	<0.01
Melville Island	0.37	0	<0.01	<0.01
Cobourg Peninsula	0.28	0.26	0	<0.01
Darwin	0.25	0.24	0.13	0

The upper triangle of the matrix contains the p-values for each pairwise value of differentiation.

**Fig. 2:**
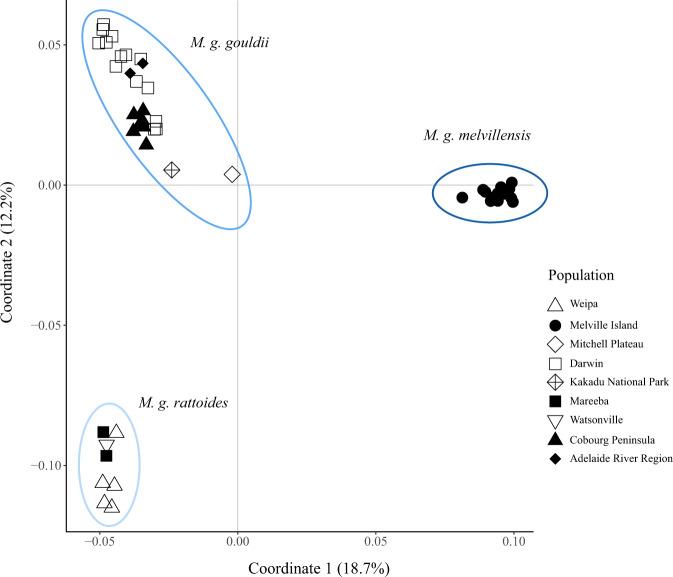
Principal coordinate plot of Nei’s genetic distance between all individual black-footed tree-rats (*Mesembriomys gouldii*). Clusters have been circled according to current subspecies taxonomy, coloured to match Fig. 3 where k = 3.

The principal coordinates plot (Fig. 2) identified three predominant clusters corresponding with known major biogeographic barriers that separate the main regions of the species’ range: Queensland and the Northern Territory (Carpentarian Gap), and the mainland and Melville Island (Beagle / Van Diemen Gulf). The first axis of the principal coordinates plot accounted for 18.7% of the total genomic variation and separated the Melville Island population from the mainland populations, with the second axis accounting for 12.2% of total genomic variation and separated the Queensland localities from the mainland Northern Territory (Fig. 2).

**Fig. 3:**
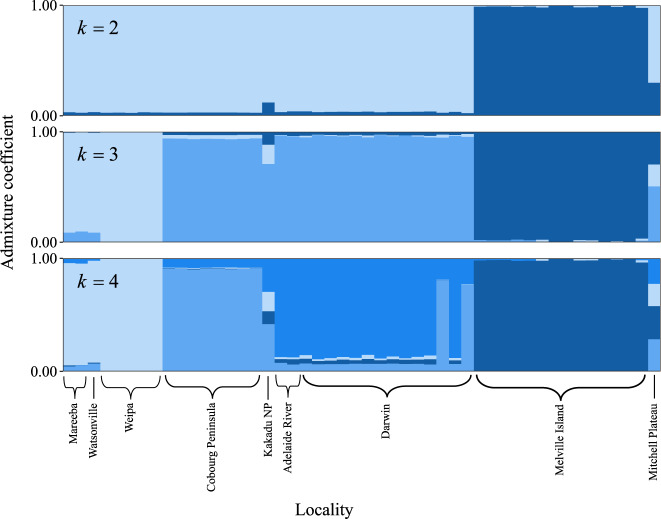
Genetic clustering in the black-footed tree-rat (*Mesembriomys gouldii*) as inferred from the *tess3r* model. Population structure is shown in four panels with individual admixture coefficients when the number of ancestral genomic clusters (*k*) identified is increased from two to four (top to bottom).

The cross-entropy plot from the analysis of genomic structure showed decreasing cross-validation scores with increasing values of *k*, consistent with a hierarchical population structure and high *F*_ST_ among sampled populations, although there were large improvements in the cross-validation score between *k* = 1 and *k* = 2, and *k* = 3 and *k* = 4 (Supplementary Material Fig. S2). Since the additional population genomic structure with each increase in *k* was nested within the previously identified clusters, we considered it informative to present the clustering results from incremental increases in *k* from 2 to 4. Visualisation of the admixture coefficients for *k* = 2 showed a separation of the Melville Island population from the mainland populations, while *k* = 3 showed a separation of the Queensland and Northern Territory populations. At *k* = 4 the Cobourg Peninsula was separated from Darwin (Fig. 3).

### Demographic history and trends in effective population size

The unrooted maximum likelihood tree inferred by TreeMix illustrated topology was largely concordant with population structuring (Supplementary Material Fig. S4) illustrated by the PCoA (Fig. 2) and hierarchical population structuring implemented in the ‘tess3r’ package (Fig. 3). This topology did not substantially change with the addition of migration events, with the optimal number of migrations chosen when m = 2 (Supplementary Material Fig. S4). Geographic localities tended to be grouped together with populations isolated by biogeographic barriers, such as Mitchell Plateau separated by the Ord Arid Region and Weipa separated by the Carpentarian Gap (Fig. 1). Geographically isolated populations showed higher levels of genetic drift, indicated by the length of the horizontal branches on the inferred tree (proportional to the amount of genetic drift that has occurred since a population became isolated). Despite m = 2 being above the 99.8% threshold, the low weights of the proposed migration events between Melville Island and both Weipa and the Adelaide River Region provide little support for contemporary gene flow.

**Fig. 4:**
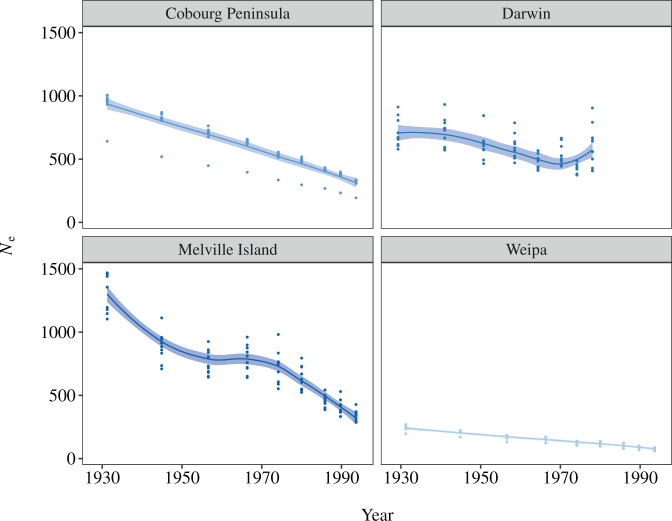
Estimated effective population size trajectories. Recent effective population size (*N*_e_) trajectories were inferred using SNeP for populations of the black-footed tree-rat (*Mesembriomys gouldii*). Colours match those used in Fig. 3 (at *k* = 4).

Investigation of historical demographics using *SNeP* suggested that most populations of the black-footed tree-rat have been experiencing declines in *N*_e_ for much of the past century (Fig. 4). The widely distributed sampling localities of Weipa, Melville Island, and the Cobourg Peninsula all showed relatively consistent patterns of decline, with the Melville Island population showing the steepest trajectory of decline (but also the largest *N*_e_). The population of black-footed tree-rats in Darwin appears to have been somewhat more stable than other areas over the past century. The final estimate for Darwin showed a potential increase in population size; however, a lack of very recent sampling (i.e., last 5–10 years) in this locality prevented estimation of post-1980 *N*_e_ values.

### Identifying population-level contributions to species genomic diversity

While the Darwin population had the highest allelic richness (1.46) of all localities (Table 3), its contribution to the overall allelic richness of the species was the lowest (0.01). Conversely, Melville Island had relatively low allelic richness (1.33) but made the greatest contribution to overall allelic richness (0.08). The Weipa population had both a low allelic richness and made just a small contribution to overall allelic richness (0.03). These results broadly agree with the principal coordinate analysis and the genomic structure results, suggesting that there is redundancy in genomic diversity among the mainland populations/subspecies, whereas gene flow between Melville Island and the mainland has been much more limited.

**Table 3:** Allelic richness (AR) of four populations of the black-footed tree-rat (*Mesembriomys gouldii*), and the contribution that each group makes to the total allelic richness of the species (i.e., where all genotyped individuals are considered one ‘population’).

	Weipa	Melville Island	Darwin	Cobourg Peninsula
Mean AR	1.28	1.33	1.46	1.42
SD	0.43	0.42	0.41	0.44
AR contribution	0.03	0.08	0.01	0.05

**Fig. 5:**
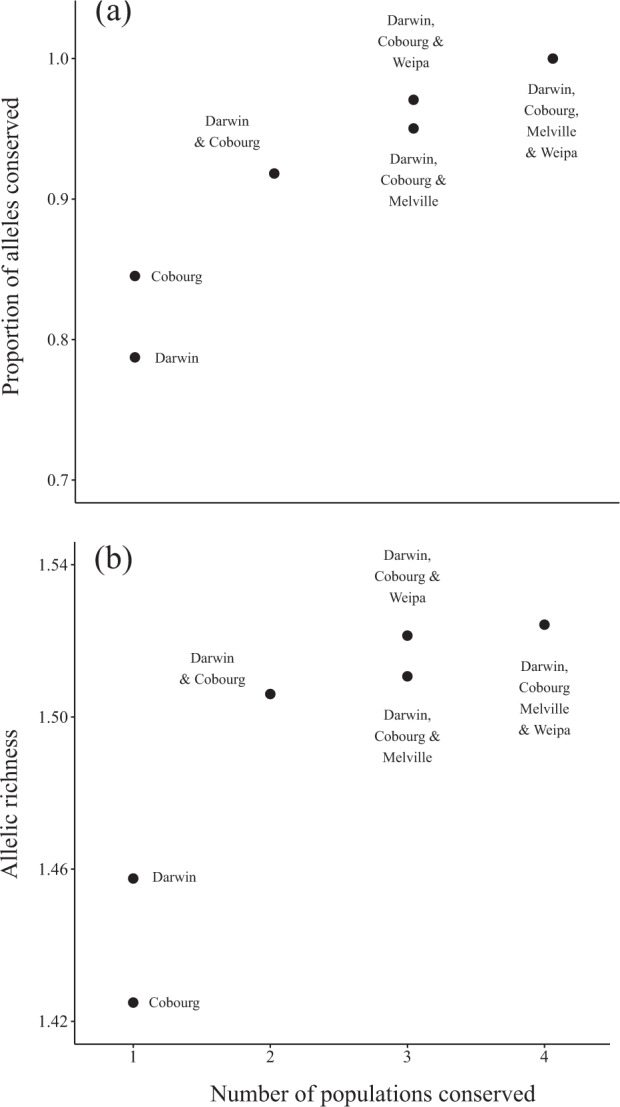
Conservation of genetic diversity across different combinations of populations. Impacts of conserving differing numbers of populations for the black-footed tree-rat (*Mesembriomys gouldii*) on (**a**) the proportion of total alleles conserved relative to the entire dataset and (**b**) allelic richness of all conserved individuals. The number of populations conserved in each scenario corresponds with those in Table S2. Where more than one population was identified as an optimal choice for conservation by the iterative marxan analysis, both scenarios are presented and the relevant populations are shown and labelled.

Given an equal cost for conserving each planning unit, marxan suggested that the Darwin population (72% of iterations) was the single most effective region to conserve for allelic diversity (representing ~81% of the alleles detected in the species), although the conservation benefit of conserving the Cobourg Peninsula population (28% of iterations) was quite similar (~75% of alleles detected in the species) (Supplementary Material Table S3; Fig. 5). If two populations were to be conserved, Cobourg Peninsula and Darwin were always selected (Supplementary Material Table S3). If three populations were conserved, Darwin and Cobourg Peninsula were always selected, with Melville Island almost always (98% of iterations) selected. Conserving the Darwin and Cobourg Peninsula populations ensured that 92% of alleles are retained, with rapidly diminishing returns as additional populations were added to the hypothetical reserve system, although our sampling in many areas was limited. Conserving just one of Darwin of the Cobourg Peninsula resulted in the loss of about 15 to 20% of alleles, with a similar trend in the contribution to allelic richness (significantly lower allelic richness if just a single population was conserved).

## Discussion

Our analyses demonstrate that the black-footed tree-rat shows substantial levels of genomic differentiation among populations across northern Australia, with the Queensland and Melville Island populations most strongly differentiated from the Kimberley and Northern Territory mainland populations. To conserve a robust amount of genomic diversity (e.g., >90% of alleles) in this declining species, the conservation of at least two mainland populations is required, with prioritisation analyses suggesting that the Cobourg Peninsula and Darwin populations are of greatest value, although further sampling of populations in Queensland and Western Australia is sorely needed. Additionally, our analysis of the trajectory of effective population sizes over the past century suggests that many populations of the black-footed tree-rat have potentially been declining for at least the last century. Much of the ecological survey data that have been used to identify small mammal declines in northern Australia come from the past three decades (Woinarski et al. 2001; Davies et al. 2018) and longer-term context for these declines has been lacking. Inference of longer-term demographic patterns from genomic data has value in helping understand the recent pattern of small mammal decline and regional geographic heterogeneity in these patterns.

### Population genomic structure

Our principal coordinate plot and analysis of differentiation patterns supports the previous subspecific circumscriptions for the black-footed tree-rat, with populations in Queensland (*M. g. rattoides*) and on Melville Island (*M. g. melvillensis*) displaying a high level of historical isolation from the rest of the mainland populations (*M. g. gouldii*). The population genomic structure of the black-footed tree-rat broadly aligns with other widespread mammal species across northern Australia, such as the brush-tailed rabbit-rat (von Takach et al. 2021) and northern quoll (von Takach et al. 2022), which also show genomic patterns consistent with 2–4 high-level evolutionary lineages. Similarly to the brachyotis group of rock wallabies (Potter et al. 2014), major biogeographic barriers separating Queensland, the Northern Territory, Western Australia, and various islands (e.g., the Tiwi Islands and Groote Eylandt) tend to define genomic structure both within and among species. These barriers have also been implicated in shaping species composition and distribution of a range of non-mammalian taxa, including eucalypts and other plant genera (Bowman et al. 2010), toadlets (*Uperoleia* spp.) (Catullo et al. 2014), and butterflies (*Nesolycaena* spp.) (Bowman et al. 2010). Together, these findings highlight that treating the black-footed tree-rat’s multiple evolutionary lineages as a single entity for conservation management is likely to result in the loss of large amounts of genomic diversity and adaptive potential. The development of strategic frameworks for conservation management that adequately consider patterns of population genomic structure is necessary to ensure that the bulk of unique genomic diversity is conserved.

While conservation units can and have been defined in myriad ways (Palsbøll et al. 2007; Funk et al. 2012), recent work tends to categorise conservation units into evolutionarily significant units, management units, and adaptive units (Barbosa et al. 2018). Evolutionarily significant units can be defined using all loci, management units can be defined using neutral loci, and adaptive units can be defined using putatively adaptive loci. Here, we used a dataset composed of primarily neutral loci with which we could identify three to four management units. However, we suggest that further sampling in the eastern and western portions of the species distribution would likely result in the presence of five or six management units, as these parts of the species distribution are almost certainly demographically independent with contemporary gene flow restricted by various threatening processes and extirpations. Further sampling may also help to enable the identification, and geographic circumscription, of adaptive units.

### Conserving genomic diversity

Analyses of genomic diversity within populations, and contributions to diversity across populations, provide data to inform different conservation planning scenarios. First, we identified the Weipa population as having particularly low genomic diversity (heterozygosity and allelic richness). When paired with that population’s low and apparently declining *N*_e_, the population is a candidate for genetic management. We suggest that an initial action should be further survey and quantification of genomic diversity in the Queensland Cape York region around Weipa, with a view to assessing the need for genetic management following further data generation. However, we note the apparent decline in *N*_e_ across the other populations sampled and suggest future demographic and genetic monitoring as priorities.

Prioritisation analyses identified the locations that contribute the greatest amount to genomic diversity in the sampled populations of the species. This provides information that can be used to develop formal conservation management strategies, forecast the likely loss of genomic diversity if individual populations become extinct, and help identify combinations of populations that, as sources for reintroductions, best represent the full genomic diversity of the species. These analyses suggest that the populations most important for the conservation of black-footed tree-rat genomic diversity were those across the mainland of the Northern Territory, followed by those in Queensland or Melville Island. While the populations around Darwin and on the Cobourg Peninsula are clearly important for conservation purposes, sampling of other remnant populations across the species’ distribution might alter the results of these analyses by uncovering geographic areas containing additional genomic diversity. For example, based on our current results, conserving just the Darwin and Cobourg populations would meet our 90% target for genomic diversity; however, the loss of black-footed tree-rats from Melville Island and in Queensland would result in the loss of two subspecies (as currently described). Further analysis after additional sampling has taken place may reduce the relative importance of the Darwin and Cobourg populations, however, this is uncertain. Importantly, our prioritisation confirms that the Cobourg Peninsula should be recognised as an important reservoir of genomic diversity for mammal species in northern Australia (von Takach et al. 2021), providing suitable habitat for a range of threatened and declining mammal species (von Takach et al. 2020a, 2020b).

### Implications for conservation management

Population genomic data can be used to inform conservation management and prioritise conservation actions and resources in a variety of ways, such as identifying areas with high levels of inbreeding or low adaptive capacity (Andersen et al. 2004), investigating patterns of connectivity and adaptive divergence among populations (Sandoval-Castillo et al. 2018), demonstrating results of, and identifying source populations for, translocations (Ottewell et al. 2014; Rick et al. 2019), and estimating historical and contemporary effective population sizes and trajectories (von Takach et al. 2022). While detailed population genomic data is still unavailable for most Australian rodents, there are a small number of recent studies that have actively applied such data to conservation practice. For example, genomic data has been used to guide strategies around translocation and supplementation of greater stick-nest rat (*Leporillus conditor*) populations (White et al. 2020), and a recent study of the brush-tailed rabbit-rat used genomic data to inform management actions aimed at conserving broad-scale genomic diversity across divergent lineages (von Takach et al. 2021).

One outcome of our demographic investigation was the apparent different trajectory of the black-footed tree-rat population in Darwin, relative to less-developed areas of the species’ range. Ecological surveys have documented that the black-footed tree-rat has thus far managed to persist in and around many remnant patches of native vegetation in the urban and rural areas of Greater Darwin (Price et al. 2005), likely due to habitat conditions or high productivity of the Darwin region mediating various threatening processes and/or bolstering population resilience (Scheele et al. 2017; von Takach et al. 2020a, 2020b). This is despite ongoing development for housing, horticulture and other industries leading to increasingly small and isolated habitat patches that are surrounded by intensive land uses or highly modified urban landscapes. The impacts of this continued clearing and modification on the population persistence, population genomic health, and population connectivity of this threatened subspecies are uncertain, and additional research into the fine-scale patterns of gene flow within and among remaining habitat patches, in the context of urban barriers, would be of substantial help to urban planners and development assessment/approval processes. Importantly, the Darwin region contains a population of black-footed tree-rats with relatively high genomic diversity that could act as an appropriate source for potential translocations elsewhere – a strategy that may prove valuable with the ongoing development in the region.

In regions outside of Darwin, temporal analysis of trapping data over recent decades has shown severe population declines in black-footed tree-rats and many other mammal species (Woinarski et al. 2010, 2014; Davies et al. 2018), broadly reflecting the trajectories that we inferred from genetic data. Currently, very little active conservation management occurs across northern Australia, with major limitations in ecological knowledge, funding, and conflicting ideologies preventing effective action being taken at landscape scales, even within many national parks and other protected areas. On Melville Island, broad-scale actions such as lethal control of feral cats and introduced herbivores (feral horses and water buffalo) have potential to improve population trajectories (Penton et al. 2021), and further research into the efficacy of such management for threatened species including the black-footed tree-rat and brush-tailed rabbit-rat would be of value in both this region and on the Cobourg Peninsula.

Conservation actions that may benefit the black-footed tree-rat include targeted early season prescribed burning, to prevent high-intensity late dry season fires that reduce habitat integrity (e.g., retention of hollow bearing and fruiting shrubs), and greater retention of old growth vegetation (i.e., 4 years at unburnt) in close proximity to known populations (von Takach et al. 2020a, 2020b; Radford et al. 2021; von Takach et al. 2022). Conserving genetic diversity in a captive breeding program could also be pursued, as has been done for other Australian rodents (Lambert et al. 2016; Abicair et al. 2020), to supplement wild populations with captive-reared individuals or act as an insurance population. While there is one such program for black-footed tree-rats in the Northern Territory, its geographic focus is on the Darwin region, and a broader program could be established that incorporates the knowledge of multiple evolutionary lineages and our understanding of their geographic spread and genomic diversity. This would also provide opportunities for future translocations (e.g., predator free islands or fenced reserves) across northern Australia if such an avenue was pursued. While the patchy distribution of the black-footed tree-rat across various land tenures poses a challenge for in-situ conservation efforts, the identification of remnant populations across northern Australia, with appropriate local fire and feral animal management activities, will likely be crucial to the persistence of the species.

### Future directions and considerations

While the black-footed tree-rat persists in parts of Western Australia and north Queensland, little is known of these populations. In Western Australia, the species appears to be very rare, and possibly at high risk of local extinction, with very few confirmed records in the last 40 years. As such, obtaining demographic and genomic data from these regions should be a priority. Genomic investigation of populations in Western Australia will help clarify their level of differentiation from, and enable comparison of genomic diversity with, the geographically distant Northern Territory populations. Further, the relative importance of feral cat predation, feral herbivore disturbance, and frequent or high-intensity fires on population persistence across the species distribution is largely uncertain. While it appears as though some populations can persist in the presence of one or more of these threats, the degree to which population demography, dispersal processes, and genetic diversity are impacted is still unknown. Critically, there is a growing body of evidence suggesting that rapid and intensive management of mammal species is required across large expanses of northern Australia (Woinarski et al. 2011; Davies et al. 2017; von Takach et al. 2020a, 2020b; Stobo-Wilson et al. 2020); however, inadequate funding and resources are preventing conservation agencies from adequately stemming declines and extinctions. Such problems reflect poorly on Australia’s broader commitment to biodiversity conservation, which is already coming under increasing scrutiny (Wintle et al. 2019). Conserving the genetic diversity of mammal species across northern Australia requires management actions targeted towards ensuring the persistence of large and stable populations in multiple geographic regions. Without the resources necessary to undertake such management actions, we will likely continue to monitor these species as their spiral towards extinction continues.

## Supplementary information

Supplemental Material

## Data Availability

All raw sequencing data have been uploaded to the Oz Mammal Genomics Initiative data portal (https://data.bioplatforms.com/organization/about/bpa-omg) (dataset ID 102.100.100/52627). All bioinformatics and R scripts, sample metadata, output and log files, and filtered and unfiltered genotypes have been uploaded to the Dryad Digital Repository (10.5061/dryad.sf7m0cg9w).
