# Supplementary material for: Population genomics and conservation management of the threatened black-footed tree-rat (Mesembriomys gouldii) in northern Australia
Source: Heredity (Edinb). 2023 Mar 10;130(5):278–88. doi: 10.1038/s41437-023-00601-0 (PMC10162988; doi:10.1038/s41437-023-00601-0)
Supplement: Supplementary file 1 — Supplemental Material [file 41437_2023_601_MOESM1_ESM.docx]

**Supplementary Material**

**Population genomics and conservation management of the threatened black-footed tree-rat (*Mesembriomys gouldii*) in northern Australia**

Brenton von Takach, Holly Sargent, Cara E. Penton, Kate Rick, Brett P. Murphy, Georgina Neave, Hugh F. Davies, Brydie M. Hill, Sam C. Banks

Table S1. Listing status of all black-footed tree-rat (*Mesembriomys gouldii*) subspecies under Australian state, territory and federal legislative acts.

| Subspecies | Western Australia | Northern Territory | Queensland | Federal |
| --- | --- | --- | --- | --- |
| *M. g. gouldii* | Endangered | Endangered | - | Endangered |
| *M. g. melvillensis* | - | Vulnerable | - | Vulnerable |
| *M. g. rattoides* | - | - | Least concern | Vulnerable |

Table S2. Filtering steps used to identify a set of informative and statistically independent genome-wide single nucleotide polymorphisms (SNPs) for population genomic analysis of the black-footed tree-rat (*Mesembriomys gouldii*).

| Step/filter | SNPs retained | Notes |
| --- | --- | --- |
| SNP calling (ANGSD) | 188,649 | minMapQ 20; minQ 20; SNP_pval 1e-5; minInd 20; setMinDepthInd 10; setMinDepth 200; setMaxDepth 13350; geno_minDepth 10; geno_maxDepth 150; postCutoff 0.98 |
| call rate | 11,382 | retained if called in > 90% samples |
| minor allele count | 11,005 | retained if MAC ≥ 3 |
| observed heterozygosity | 10,837 | retained if Ho ≤ 0.6 |
| linkage disequilibrium | 4,764 | retained if correlation < 0.5 within a sliding window of 100,000 bp. Reducing the sliding window size to 10,000 bp only retains an additional 29 SNPs. |
| outlier identification | 4,764 | No outlier loci identified using two methods (Frichot & François, 2015; Whitlock & Lotterhos, 2015) |
| sex-linked | 4,764 | retained if not significantly sex-biased (Y-linked, X-linked or XY gametolog) |


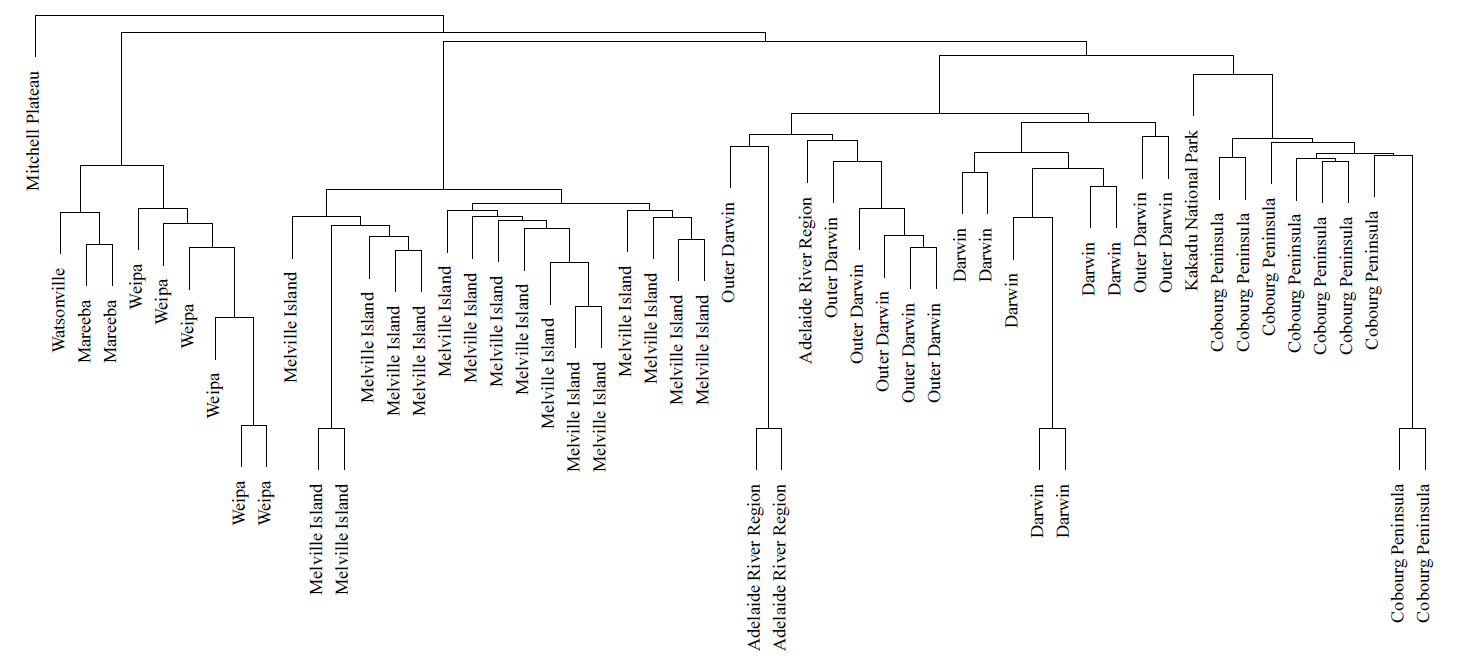


Figure S1. Hierarchical clustering dendrogram representing genetic distance relationships between black-footed tree-rat (*Mesembriomys gouldii*) samples. The five technical replicates are represented by samples paired on long branches.


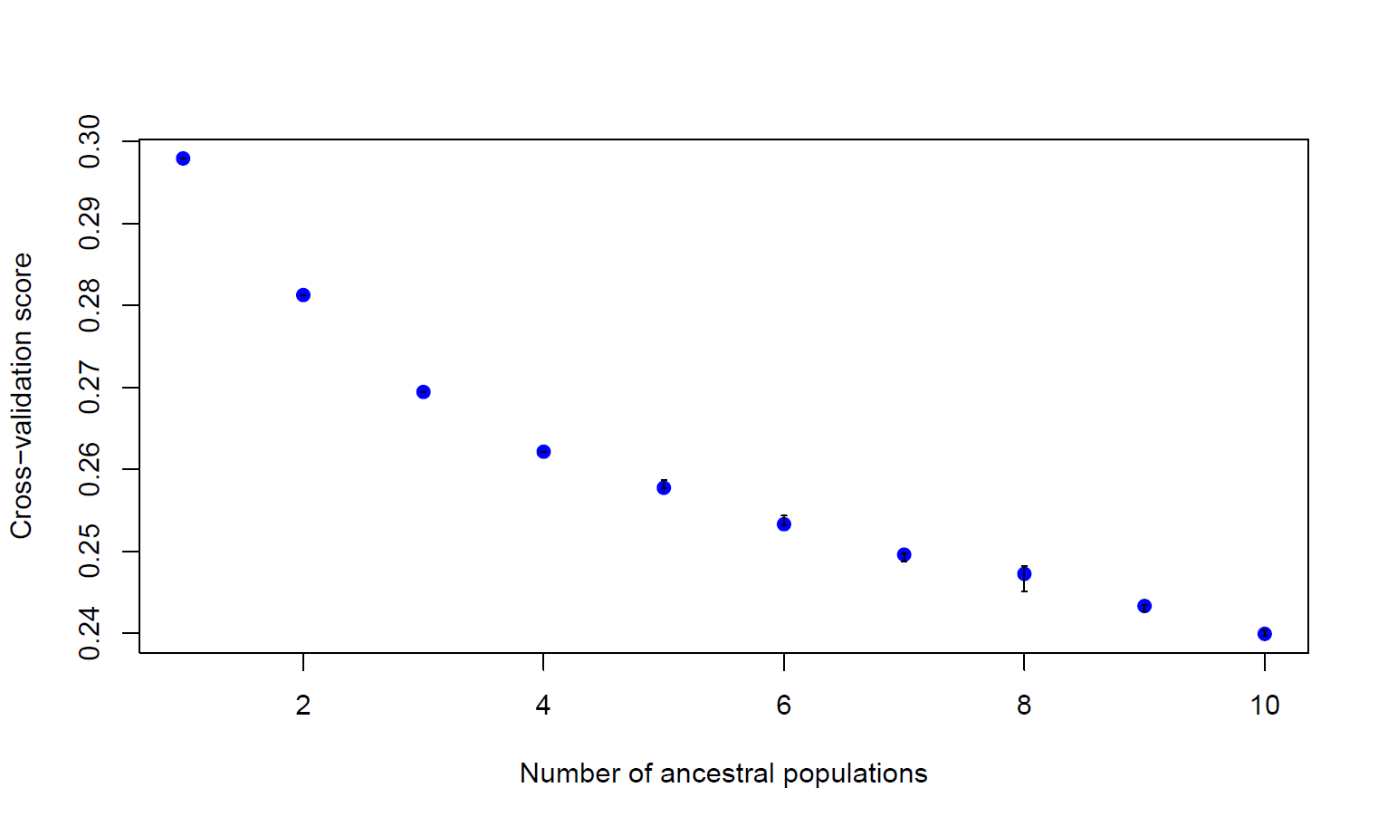


Figure S2. Cross-entropy plot used to identify hierarchical population structuring in black-footed tree-rats (*Mesembriomys gouldii*). Lower values of the cross-entropy criterion indicate a better fit to the data. A large drop in cross-entropy from the preceding point indicates that a *k* value is well-supported.


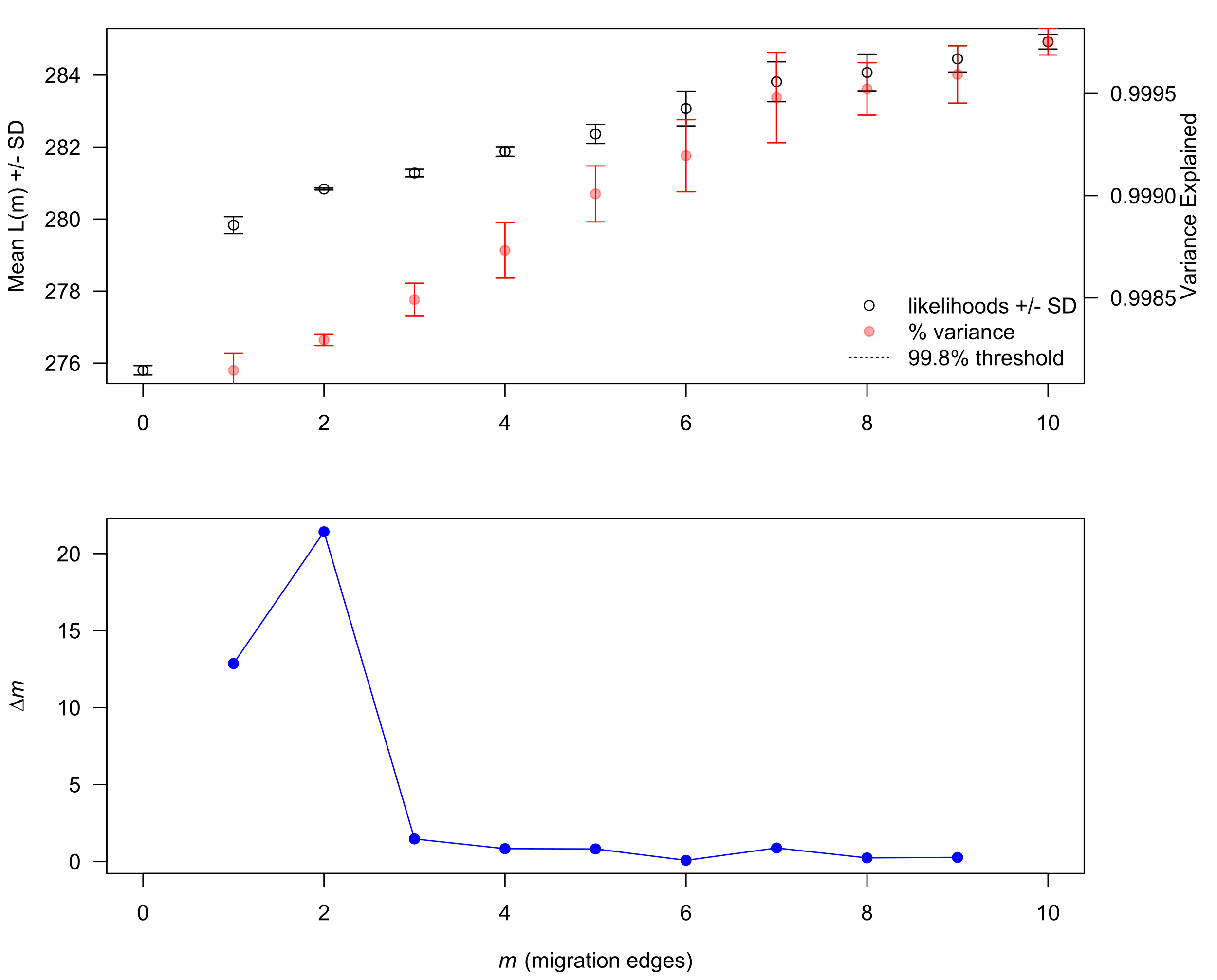


Figure S3. The mean and standard deviation (SD) across 10 iterations for the composite likelihood L(m) (left axis, black circles) and proportion of variance explained (right axis, red circles) (top); and the second-order rate of change (Δm) across values of m (bottom). Both figures are the output produced by the R package ‘OptM’.


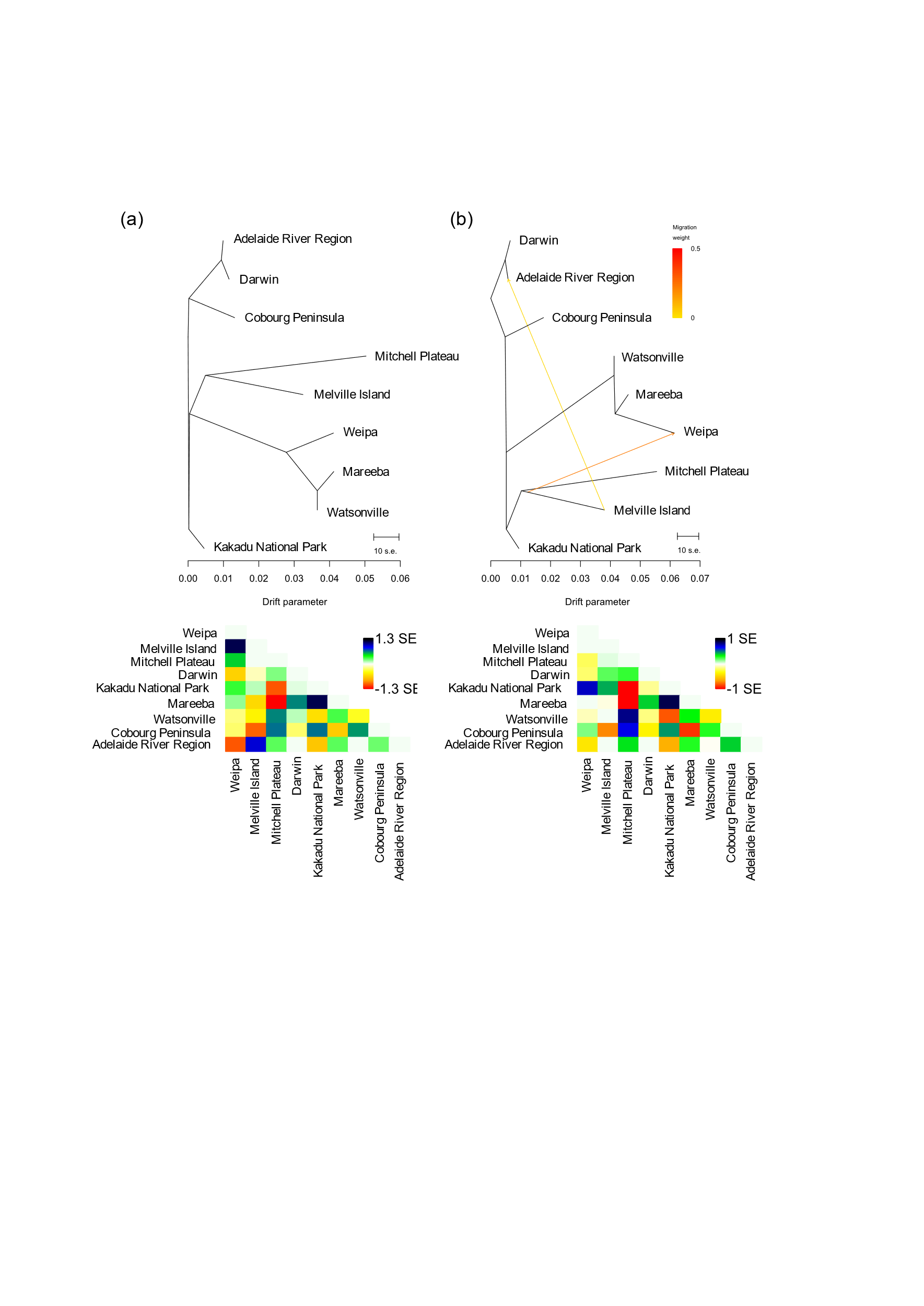


Figure S4. TreeMix unrooted maximum likelihood tree of the black-footed tree-rat (*Mesembriomys gouldii*) based on 4764 SNPs with (a) no migration events and (b) allowing two migration events. Drift parameter is shown on the x-axis with horizontal branch lengths proportional to the amount of genetic drift that has occurred on the branch. The scale bar shows 10× the average standard error of the entries in the sample covariance matrix. The residual fit for each tree is also illustrated with large, positive residuals (blue/black colours) indicating population pairs that are more closely related to each other than suggested by the tree and may indicate potential admixture events. Migration arrows are coloured according to their weight based on the proportion of alleles in the descendent population that are derived from the ancestral population.

Table S3. Optimisation scenarios for conservation of alleles in populations of the black-footed tree-rat (*Mesembriomys gouldii*). Numbers show the proportion of times that each population was chosen in a given scenario from 100 iterations of randomly sampling four individuals per population and counting the number of alleles.

| Number of populations conserved | Weipa | Melville Island | Darwin | Cobourg Peninsula |
| --- | --- | --- | --- | --- |
| 1 | 0 | 0 | 0.72 | 0.28 |
| 2 | 0 | 0 | 1 | 1 |
| 3 | 0.02 | 0.98 | 1 | 1 |
| 4 | 1 | 1 | 1 | 1 |
